# MALDI-HRMS Imaging Maps the Localization of Skyrin, the Precursor of Hypericin, and Pathway Intermediates in Leaves of *Hypericum* Species

**DOI:** 10.3390/molecules25173964

**Published:** 2020-08-31

**Authors:** Bharadwaj Revuru, Miroslava Bálintová, Jana Henzelyová, Eva Čellárová, Souvik Kusari

**Affiliations:** 1 Center for Mass Spectrometry (CMS), Department of Chemistry and Chemical Biology (CCB), Technische Universität Dortmund, Otto-Hahn-Straße 6, 44221 Dortmund, Germany; bharat.revuru@gmail.com; 2 Institute of Biology and Ecology, Faculty of Science, Pavol Jozef Šafárik University in Košice, Mánesova 23, 041 54 Košice, Slovak Republic; miroslava.balintova@upjs.sk (M.B.); jana.henzelyova@upjs.sk (J.H.)

**Keywords:** *Hypericum*, skyrin, hypericin, naphthodianthrones, MALDI-HRMS imaging

## Abstract

*Hypericum perforatum* and related species (Hypericaceae) are a reservoir of pharmacologically important secondary metabolites, including the well-known naphthodianthrone hypericin. However, the exact biosynthetic steps in the hypericin biosynthetic pathway, vis-à-vis the essential precursors and their localization in plants, remain unestablished. Recently, we proposed a novel biosynthetic pathway of hypericin, not through emodin and emodin anthrone, but skyrin. However, the localization of skyrin and its precursors in *Hypericum* plants, as well as the correlation between their spatial distribution with the hypericin pathway intermediates and the produced naphthodianthrones, are not known. Herein, we report the spatial distribution of skyrin and its precursors in leaves of five in vitro cultivated *Hypericum* plant species concomitant to hypericin, its analogs, as well as its previously proposed precursors emodin and emodin anthrone, using MALDI-HRMS imaging. Firstly, we employed HPLC-HRMS to confirm the presence of skyrin in all analyzed species, namely *H. humifusum*, *H. bupleuroides*, *H. annulatum*, *H. tetrapterum,* and *H. rumeliacum*. Thereafter, MALDI-HRMS imaging of the skyrin-containing leaves revealed a species-specific distribution and localization pattern of skyrin. Skyrin is localized in the dark glands in *H. humifusum* and *H. tetrapterum* leaves together with hypericin but remains scattered throughout the leaves in *H. annulatum*, *H. bupleuroides*, and *H. rumeliacum*. The distribution and localization of related compounds were also mapped and are discussed concomitant to the incidence of skyrin. Taken together, our study establishes and correlates for the first time, the high spatial distribution of skyrin and its precursors, as well as of hypericin, its analogs, and previously proposed precursors emodin and emodin anthrone in the leaves of *Hypericum* plants.

## 1. Introduction

**Figure 1:**
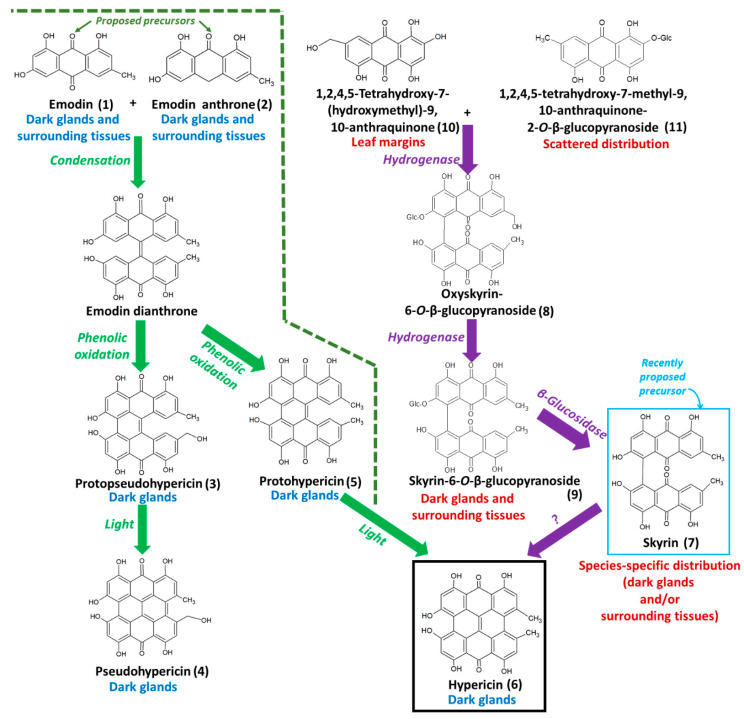
The proposed biosynthetic pathways of hypericin. The biosynthesis of hypericin and its protoforms using emodin **(1)** and emodin anthrone **(2)** as precursors is represented on the left side with green colored arrows. The localization of compounds is represented in blue color according to our previous work [7]. The biosynthesis of skyrin **(7)** and proposed hypericin **(6)** production through skyrin **(7)** as a precursor is represented on the right with violet arrows [17]. Our present work reports for the first time, the occurrence and spatial distribution of skyrin **(7)** and its precursors in *Hypericum* leaves concomitant to hypericin **(6)**, its protoforms **(3**–**5)**, as well as its previously proposed precursors emodin **(1)** and emodin anthrone **(2)**.

Secondary metabolites actively participate in a plethora of physiological activities in plants, which includes imparting stress tolerance and accessory functions, unlike primary metabolites [1]. Additionally, these plant-derived specialized metabolites exhibit a wide array of pharmacological activities, which has opened gates to explore plant communities for novel compounds [2]. Hypericaceae is a central, ethnomedicinal plant family, within which *Hypericum perforatum* has been extensively studied for its bioactive metabolites [3]. *H. perforatum*, commonly known as St. John’s wort, accumulate naphthodianthrone metabolites such as hypericin **(6)**, protohypericin **(5)**, pseudohypericin **(4)**, and phloroglucinol hyperforin possessing anti-inflammatory, antioxidant, anticancer, and antimicrobial properties, in the aerial parts of the plant, especially flowers and leaves [4,5] (compounds are numbered according to hypericin **(6)** biosynthetic pathway, see Figure 1). Besides, hypericin **(6)** has been reported as a photosensitizer and is used effectively against nonmelanoma skin cancers [4]. Transcriptomic analysis of four different *Hypericum* species revealed that biosynthesis of hypericin **(6)** is concentrated to marginal regions of the leaves, and primarily localized in the dark glands [6]. Using a combination of HPLC-HRMS and matrix-assisted laser desorption/ionization high-resolution mass spectrometry (MALDI-HRMS) imaging, we confirmed that hypericin **(6)**, along with its analogs/protoforms protohypericin **(5)** and pseudohypericin **(4)**, accumulate in the dark glands [7]. In contrast, its proposed precursor emodin **(1)**, is typically distributed both inside and outside the dark glands. In agreement with this, both Hölscher et al. (2009) and Rizzo et al. (2019) reported the accumulation of hypericin **(6)** in the dark glands, which plants develop during the placental stage [8,9]. The reason behind the evolutionarily-evolved accumulation of hypericin **(6)** in dark glands is due to its photosensitizing activities [10].

The biosynthetic pathway of hypericin initiates with the condensation of 7 molecules of malonyl-CoA and one molecule of acetyl-CoA, giving rise to emodin **(1)** and emodin anthrone **(2)** catalyzed by polyketide synthase (PKS) [11]. Subsequently, emodin **(1)** and emodin anthrone **(2)** condense to form protohypericin **(5)**, which is immediately converted to hypericin **(6)**, and this reaction is catalyzed by light [12]. Initially, Bais et al. (2003) proposed the *Hyp-1* gene product as the key enzyme involved in the biosynthesis of hypericin through dimerization of emodin **(1)** and emodin anthrone **(2)**, and phenolic oxidation to protohypericin **(5)** and hypericin **(6) [11]**. However, later Košuth et al. (2007) demonstrated that *Hyp-1* expression is higher in roots compared to aboveground parts [13]. This is contrary to the site of hypericin **(6)** accumulation in *Hypericum* plants, which is characteristically seen in aboveground parts [12,14]. Further, Košuth et al. (2010) proposed that the *Hyp-1* gene product may not be involved in hypericin **(6)** production by demonstrating that *Hyp-1* is constitutively expressed in all plant tissues irrespective of the presence of hypericin **(6)** and emodin **(1)** [12]. This assumption was further supported by X-ray crystallographic studies of Michalska et al. (2010) and Sliwiak et al. (2016) [15,16]. Thus far, the exact biosynthetic steps in the hypericin biosynthetic pathway, in particular, the key precursors and their localization in plants, remain unclear.

Recently, we proposed a novel biosynthetic pathway of hypericin, not through emodin **(1)** and emodin anthrone **(2)**, but skyrin **(7)** (Figure 1) [17]. Hypericin-containing *Hypericum* species, including *H. perforatum*, produce skyrin **(7)** and its precursors such as 1,2,4,5-tetrahydroxy-7-(hydroxymethyl)-9,10-anthraquinone **(10)**, 1,2,4,5-tetrahydroxy-7-methyl-9,10-anthraquinone-2-*O*-β-glucopyranoside **(11)**, skyrin-6-*O*-β-glucopyranoside **(9)**, and oxyskyrin-6-*O*-β-glucopyranoside **(8)**. 1,2,4,5-tetrahydroxy-7-methyl-9,10-anthraquinone-2-*O*-β-glucopyranoside **(11)** and 1,2,4,5-tetrahydroxy-7-(hydroxymethyl)-9,10-anthraquinone **(10)** combine to form the compound oxyskyrin-6-*O*-β-glucopyranoside **(8)** through hydrogenation. Later, oxyskyrin-6-*O*-β-glucopyranoside **(8)** is converted to skyrin-6-*O*-β-glucopyranoside **(9)** with another round of hydrogenation. Finally, the compound skyrin-6-*O*-β-glucopyranoside **(9)** is converted into skyrin **(7),** possibly by the β-glucosidase reaction (Figure 1) [17]. Though the genes responsible for skyrin **(7)** biosynthesis remain uncharacterized in *Hypericum* species, we recently established the skyrin-mediated production of hypericin **(6)** through our proposed biosynthetic pathway [17]. Besides, Hölzl et al. (2003) established skyrin **(7)** as a precursor of protohypericin **(5)** [18]. Remarkably, skyrin **(7)** is typically biosynthesized by fungi belonging to different species and ecological niches and reported to have antimicrobial properties [19,20]. Thus far, the role of plant-associated microorganisms such as endophytes in the production of skyrin **(7)** is a plausible, open question, particularly since native endophytes harbored in *H. perforatum* can produce hypericin **(6)** [21,22,23]. Therefore, it is essential to identify the in planta site of localization and the role of skyrin **(7)**, as well as the related intermediates leading to the production of hypericin **(6)**. This can aid in developing metabolic engineering approaches to increase the production of hypericin **(6)**. In particular, the following open questions remain unanswered:

(1) In which plant tissues is skyrin **(7)** localized and accumulated?
(2) In which tissues are the precursors of skyrin **(7)** localized and accumulated?
(3) How does the spatial distribution of skyrin **(7)** correlate with other precursors, intermediates, and the produced naphthodianthrones?

In order to answer the aforementioned questions, we employed a combination of HPLC-HRMS and matrix-assisted laser desorption/ionization high-resolution mass spectrometry imaging (MALDI-HRMS imaging). In the present study, we first confirmed the occurrence of skyrin **(7)** and its precursors in leaves of five in vitro cultivated *Hypericum* plant species concomitant to emodin **(1)**, emodin anthrone **(2)**, protohypericin **(5)**, pseudohypericin **(4)**, protopseudohypericin **(3)**, and hypericin **(6)** by HPLC-HRMS. After that, using MALDI-HRMS imaging, we visualized the distribution and dynamics of skyrin **(7)** and its precursors in the leaves in high spatial resolution, compared to hypericin **(6)** and its analogs, as well as its proposed precursors (emodin **(1)** and emodin anthrone **(2)**). In particular, both the dorsal and ventral sides of the leaves were mapped with emphasis on the dark glands, where hypericin **(6)** is localized [7], as well as the tissues surrounding the glands. Our study unravels for the first time, the occurrence, distribution, and dynamics of skyrin **(7)** and its precursors, versus the accumulation of hypericin **(6)**, its analogs, and its possible precursors in the leaves of *Hypericum* plants.

## 2. Results and Discussion

### 2.1. Detection of the Selected Phytochemicals by HPLC-HRMS

*Hypericum* species produce various anthraquinones such as hypericin **(6)**, emodin **(1)**, emodin anthrone **(2),** and their protoforms **(3**–**5)**. Among all, hypericin **(6)** is well-studied for its occurrence and spatial distribution; previous reports have established that hypericin **(6)** and its protoforms accumulate in the dark glands owing to their photosensitizing properties [10]. On the other hand, the proposed precursors, namely emodin **(1)** and emodin anthrone **(2)** accumulate both in the dark glands as well as being distributed outside the glands [7,8]. Moreover, we recently reported that skyrin **(7)** and its precursors serve as intermediates to hypericin production through another pathway, not involving emodin **(1)** or emodin anthrone **(2)** [17]. Against this background, five different *Hypericum* species, namely *H. humifusum*, *H. tetrapterum, H. annulatum, H. bupleuroides,* and *H. rumeliacum* were grown in vitro in Murashige and Skoog (MS) medium with Gamborg B5 vitamin supplements, and the leaves of the plants were extracted and subjected to selective metabolic profiling using HPLC-HRMS following our established protocol [7]. We focused on hypericin **(6)** as well as specific metabolites related to its biosynthesis, such as emodin **(1)**, emodin anthrone **(2)**, pseudohypericin **(4)**, protopseudohypericin **(3)** and protohypericin **(5)**. More importantly, we analyzed skyrin **(7)** and its precursors vis-à-vis oxyskyrin-6-*O*-β-glucopyranoside **(8)**, skyrin-6-*O*-β-glucopyranoside **(9)**, 1,2,4,5-tetrahydroxy-7-(hydroxymethyl)-9,10-anthraquinone **(10)** and 1,2,4,5-tetrahydroxy-7-methyl-9,10-anthraquinone-2-*O*-β-glucopyranoside **(11)**.

Emodin **(1)** was detected in four samples except in *H. bupleuroides* (<LOD). Our results were in accordance with previous reports where emodin was not found in the *Hypericum* species mentioned above [24,25]. Conversely, emodin anthrone **(2)** was typically either not detected or in low abundance in the samples where emodin **(1)** was detected in higher abundance. Emodin **(1)** and emodin anthrone **(2)** condenses to form an unstable compound called emodin dianthrone, which is converted to hypericin **(6)** in two subsequent steps (Figure 1). However, it has been previously reported that emodin **(1)** levels in leaves do not correlate with hypericin **(6)** accumulation [12], and the spatial distribution of emodin **(1)** is not restricted to dark glands, unlike to that of hypericin **(6)** [7]. Hence, our observed dissimilarities in the levels of these two compounds could be attributed to the fact that flux is directed towards the production of hypericin **(6)** and its analogs, pseudohypericin **(4)** and protopseudohypericin **(3)** (Table S1).

The protoforms of hypericin **(6)** like pseudohypericin **(4)**, protopseudohypericin **(3)**, and protohypericin **(5)** were detected in four analyzed *Hypericum* species, except in *H. bupleuroides.* The differential accumulation of pseudohypericin **(4)** and protopseudohypericin **(3)** in different *Hypericum* species could be due to differences in the availability of the precursor pool, similar to the pattern we observed earlier [17]. Correspondingly, protohypericin **(5)**, an immediate precursor of hypericin **(6)**, was also accumulated differentially among the analyzed species. Except for a few species, our results were in agreement with Kucharíková et al. (2016) concerning the occurrence of protohypericin [26]. Emodin dianthrone is converted into protohypericin **(5)** through phenolic oxidation [7]. Interestingly, skyrin **(7)** was also proposed as a possible precursor of protohypericin (**5**) [18]. Thus far, it could be postulated that emodin **(1)** and skyrin **(7)** could contribute their flux separately towards the synthesis of protohypericin **(5)**, and the variation observed among different species could be because of the difference in endogenous levels of precursors.

Skyrin **(7)** was detected in all five *Hypericum* species, namely *H. humifusum*, *H. bupleuroides*, *H. annulatum*, *H. tetrapterum,* and *H. rumeliacum* [17]. Intriguingly, we detected skyrin **(7)** in *H. bupleuroides* for the first time. Surprisingly, immediate precursors of skyrin **(7)**, namely skyrin-6-*O*-β-glucopyranoside **(9)** and oxyskyrin-6-*O*-β-glucopyranoside **(8)**, were not detected in any of the samples by HPLC-HRMS (i.e., <LOD), possibly because they serve as reactive intermediates in the pathway or are unstable. On the other hand, 1,2,4,5-tetrahydroxy-7-(hydroxymethyl)-9,10-anthraquinone **(10)** and 1,2,4,5-tetrahydroxy-7-methyl-9,10-anthraquinone-2-*O*-β-glucopyranoside **(11)** were found to be differentially accumulated across the species (Table S1).

Hypericin **(6)** was detected in all the analyzed samples except in *H. bupleuroides* in which skyrin **(7)** was produced. Further, emodin **(1)** was not detected in *H. bupleuroides,* although emodin anthrone **(2)** was detected. On the one hand, the presence of skyrin **(7)** coupled to the absence of hypericin **(6)** or its protoforms in *H. bupleuroides* lends possible hints that the biosynthesis of hypericin **(6)** through emodin **(1)** and related intermediates might be species-specific (Table S1). On the other hand, it is possible that skyrin production occurs in the vegetative stage, followed by its utilization for the biosynthesis of hypericin (final product of the pathway) during floral development and generation of dark glands.

### 2.2. MALDI-HRMS Imaging Reveals That Skyrin Is Localized in the Dark Glands in H. humifusum and H. tetrapterum

Selective metabolic profiling using HPLC-HRMS revealed the presence of skyrin **(7)** in the five species of *Hypericum* under investigation. Therefore, we used MALDI-HRMS imaging to map both the dorsal and ventral leaf surfaces, in high spatial resolution and with minimum sample preparation, the localization of skyrin **(7)** vis-à-vis its precursors, hypericin **(6)**, emodin **(1)**, emodin anthrone **(2)**, pseudohypericin **(4)**, protopseudohypericin **(3)**, and protohypericin **(5)**. While hypericin **(6)** and its analogs are synthesized and accumulated in the dark glands [7,8,26], hyperforin is present in translucent glands [27,28]. We analyzed both the dorsal and ventral sides of the leaves, and our critical focus was on the dark glands where hypericin **(6)** accumulates. Primarily, our emphasis was to determine the spatial distribution of skyrin **(7)** and its precursors. Localization of these compounds could help to understand whether skyrin **(7)** and its metabolic precursors are explicitly localized to the dark glands along with hypericin **(6)** or distributed throughout the leaves. Furthermore, HRMS^2^ was also performed during MALDI-HRMS imaging on the selected portion of leaves, both from the dorsal and ventral sides, to reconfirm the identity of compounds in the imaging mode following our previously established method [7,17]. We segregated and interpreted the results centering primarily on the distribution of skyrin **(7)**. 

**Figure 2:**
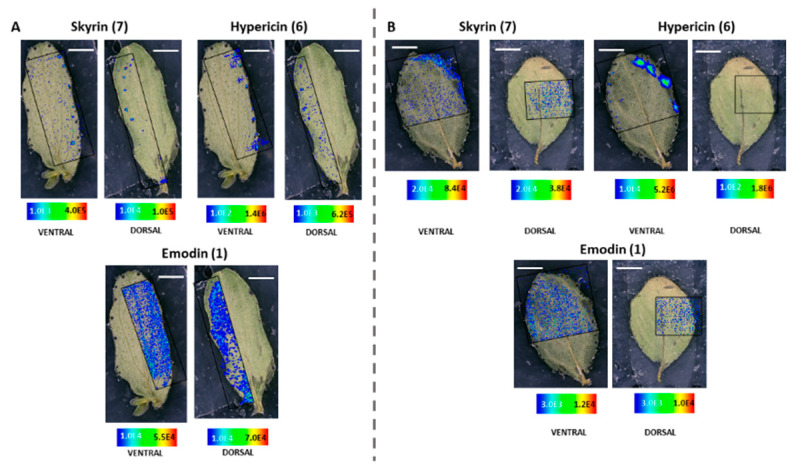
Selected ion images depicting the localization of skyrin **(7)** (*m***/***z* 537.086; [M − H]^−^; ±2 ppm; experimental), hypericin **(6)** (*m*/*z* 503.074; [M − H]^−^; ±2 ppm; experimental) and emodin **(1)** (*m*/*z* 269.045; [M − H]^−^; ±2 ppm; experimental). (**A**) Occurrence and localization of skyrin **(7)**, hypericin **(6)**, and emodin **(1)** mapped from the ventral and dorsal surfaces of *H. humifusum* leaves. (**B**) Occurrence and localization of skyrin **(7)**, hypericin **(6)**, and emodin **(1)** mapped from the dorsal and ventral surfaces of *H. tetrapterum* leaves. The assigned scale bar represents 1 mm. Black insert depicts the scanned area in each case.

**Figure 3:**
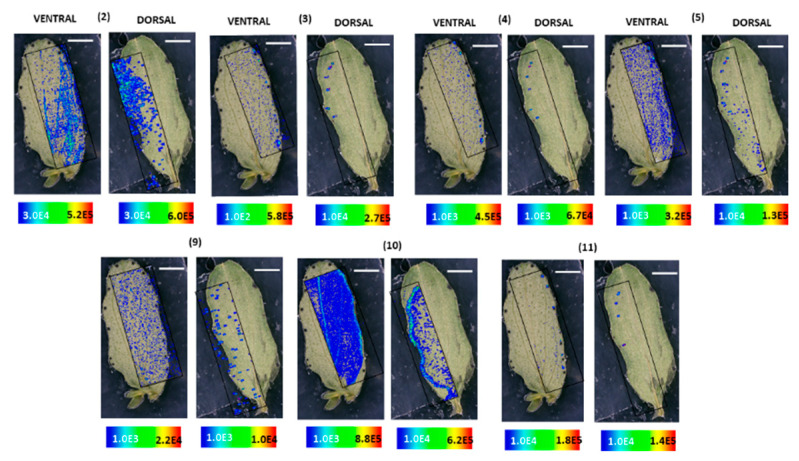
Localization and distribution of compounds in *H. humifusum* leaves. Emodin anthrone **(2)** (*m*/*z* 255.062; [M − H]^−^; ±2 ppm; experimental), protopseudohypericin **(3)** (*m*/*z* 521.082; [M − H]^−^; ±2 ppm; experimental), pseudohypericin **(4)** (*m*/*z* 519.066; [M − H]^−^; ±2 ppm; experimental), protohypericin **(5)** (*m*/*z* 505.091; [M − H]^−^; ±2 ppm; experimental), skyrin-6-*O*-β-glucopyranoside **(9)** (*m*/*z* 699.141; [M − H]^−^; ±2 ppm; experimental), 1,2,4,5-tetrahydroxy-7-(hydroxymethyl)-9,10-anthraquinone **(10)** (*m*/*z* 301.034; [M − H]^−^; ±2 ppm; experimental), 1,2,4,5-tetrahydroxy-7-methyl-9,10-anthraquinone-2-*O*-β-glucopyranoside **(11)** (*m*/*z* 447.097; [M − H]^−^; ±2 ppm; experimental). The assigned scale bar represents 1 mm. Black insert depicts the scanned area in each case.

MALDI-HRMS imaging revealed that in *H. humifusum* and *H. tetrapterum* leaves, skyrin **(7)** was typically localized in the dark glands. The similarity in the pattern of skyrin **(7)** distribution in the two *Hypericum* species was noted. In *H. humifusum* leaves, skyrin **(7)** was found to localize in high abundance in the dark glands mapped from both the dorsal and ventral leaf surfaces, similar to that of hypericin **(6)** (Figure 2A). Emodin **(1)** was distributed throughout the leaves as observed both from the dorsal and ventral surfaces, although with slightly higher abundance at the leaf margins (Figure 2A). The present results corroborate our earlier observation [7], where emodin **(1)** was found to be distributed throughout the leaves. These results indicate that emodin might be channeled to various regions of leaves for performing other plausible physiological functions such as feeding deterrent and anti-pathogenic activities [29,30]. Besides, the distribution pattern of emodin anthrone **(2)** was quite similar to that of emodin **(1)** (Figure 3). Pseudohypericin **(4)**, protopseudohypericin **(3)**, and protohypericin **(5)** accumulated in the dark glands along with hypericin **(6)** and skyrin **(7)** (Figure 3). Interestingly, the two anthraquinone derivative precursors of skyrin **(7)**, vis-à-vis 1,2,4,5-tetrahydroxy-7-(hydroxymethyl)-9,10-anthraquinone **(10)** and 1,2,4,5-tetrahydroxy-7-methyl-9,10-anthraquinone-2-*O*-β-glucopyranoside **(11)** (Figure 1), were distributed at the leaf margins and in the dark glands, respectively. Moreover, a high abundance of the anthraquinone derivative 1,2,4,5-tetrahydroxy-7-(hydroxymethyl)-9,10-anthraquinone **(10)** was observed throughout the leaf margins (Figure 3). In particular, the colocalization of 1,2,4,5-tetrahydroxy-7-methyl-9,10-anthraquinone-2-*O*-β-glucopyranoside **(11)** with hypericin **(6)** in the dark glands opens up the question whether the glucopyranoside moiety might play a role in the translocation of hypericin pathway intermediates between the dark glands and surrounding tissues. Our results corroborate our previous observation where we could group skyrin **(7)** and analogs with hypericin **(6)** by principal component analysis (PCA), which further lent evidence to skyrin **(7)** being a precursor of hypericin **(6)** [17].

**Figure 4:**
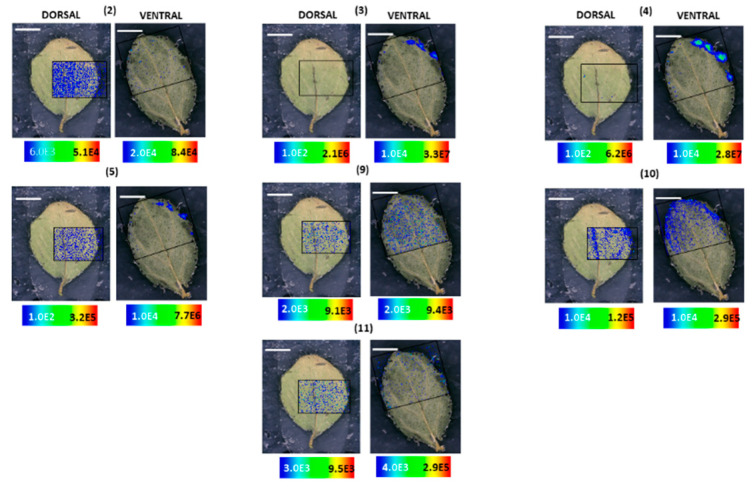
Localization and distribution of compounds in *H. tetrapterum* leaves. Emodin anthrone **(2)** (*m*/*z* 255.062; [M − H]^−^; ±2 ppm; experimental), protopseudohypericin **(3)** (*m*/*z* 521.082; [M − H]^−^; ±2 ppm; experimental), pseudohypericin **(4)** (*m*/*z* 519.066; [M − H]^−^; ±2 ppm; experimental), protohypericin **(5)** (*m*/*z* 505.091; [M − H]^−^; ±2 ppm; experimental), skyrin-6-*O*-β-glucopyranoside **(9)** (*m*/*z* 699.141; [M − H]^−^; ±2 ppm; experimental), 1,2,4,5-tetrahydroxy-7-(hydroxymethyl)-9,10-anthraquinone **(10)** (*m*/*z* 301.034; [M − H]^−^; ±2 ppm; experimental), 1,2,4,5-tetrahydroxy-7-methyl-9,10-anthraquinone-2-*O*-β-glucopyranoside **(11)** (*m*/*z* 447.097; [M − H]^−^; ±2 ppm; experimental). Assigned scale bar represents 1 mm. Black insert depicts the scanned area in each case.

The results obtained with *H. tetrapterum* were somewhat similar to *H. humifusum*. In *H. tetrapterum*, the dark glands were present on the ventral surface of the leaves, thereby allowing higher accessibility and better mapping of all the compounds with MALDI-HRMS imaging compared to the dorsal surface (Figure 2B and Figure 4). As expected, a high accumulation of hypericin **(6)** was observed in the dark glands measured from the ventral surface (Figure 2B). Similarly, intensities of skyrin **(7)** were abundant in the dark glands. Emodin **(1)** was distributed throughout the leaves. The analogs of hypericin **(6)** vis-à-vis pseudohypericin **(4)**, protopseudohypericin **(3)**, and protohypericin **(5)** were found to accumulate in the dark glands, matching the localization of hypericin **(6)** (Figure 4). Remarkably, skyrin-6-*O*-β-glucopyranoside **(9)** was also localized in the same tissue regions where skyrin **(7)** accumulated. Anthraquinone compounds 1,2,4,5-tetrahydroxy-7-(hydroxymethyl)-9,10-anthraquinone **(10)**, 1,2,4,5-tetrahydroxy-7-methyl-9,10-anthraquinone-2-*O*-β-glucopyranoside **(11)** were detected in low abundances. Interestingly, the relative abundance of 1,2,4,5-tetrahydroxy-7-(hydroxymethyl)-9,10-anthraquinone throughout leaf margins was found to be similar to the distribution in *H. humifusum* (Figure 4). Taken together, we could successfully visualize and map the spatial distribution of skyrin **(7)** and its precursors compared to other intermediates in the hypericin **(6)** biosynthetic pathway, in *H. humifusum* and *H. tetrapterum* plants.

### 2.3. MALDI-HRMS Imaging Reveals A Scattered Distribution of Skyrin in H. annulatum, H. bupleuroides, and H. rumeliacum

**Figure 5:**
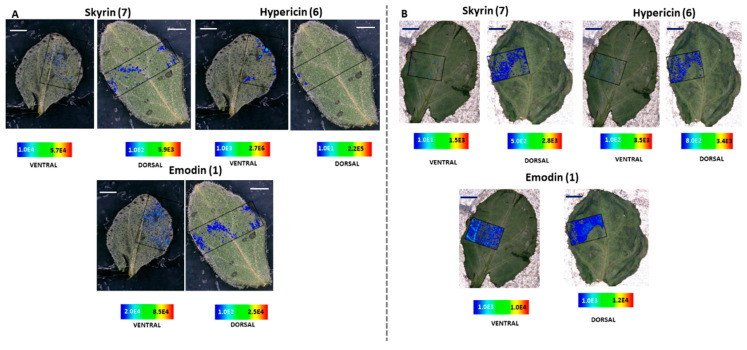
Selected ion images depicting the localization of skyrin **(7)** (*m*/*z* 537.086; [M − H]^−^; ±2 ppm; experimental), hypericin **(6)** (*m*/*z* 503.074; [M − H]^−^; ±2 ppm; experimental), and emodin **(1)** (*m*/*z* 269.045; [M − H]^−^; ±2 ppm; experimental). (**A**) Occurrence and localization of skyrin **(7)**, hypericin **(6)**, and emodin **(1)** mapped from the ventral and dorsal sides of *H. annulatum* leaves. (**B**) Occurrence and localization of skyrin **(7)**, hypericin **(6)**, and emodin **(1)** mapped from the dorsal and ventral surfaces of *H. bupleuroides* leaves. The assigned scale bar represents 1 mm. Black insert depicts the scanned area in each case.

**Figure 6:**
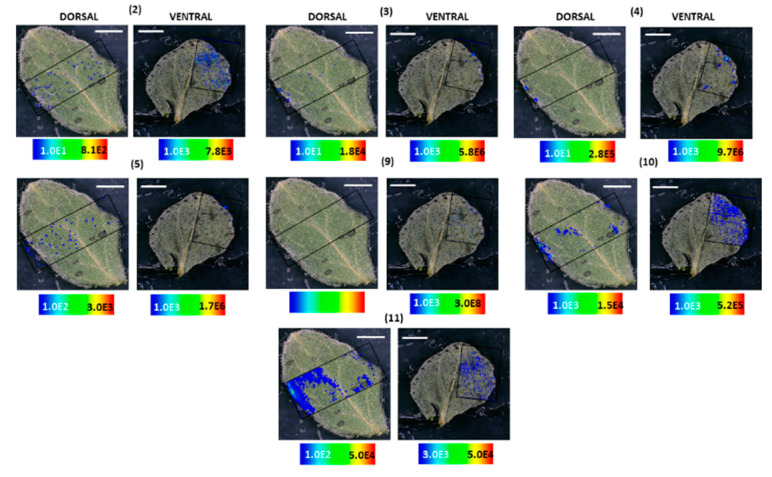
Localization and distribution of compounds in *H. annulatum* leaves. Emodin anthrone **(2)** (*m*/*z* 255.062; [M − H]^−^; ±2 ppm; experimental), protopseudohypericin **(3)** (*m*/*z* 521.082; [M − H]^−^; ±2 ppm; experimental), pseudohypericin **(4)** (*m*/*z* 519.066; [M − H]^−^; ±2 ppm; experimental), protohypericin **(5)** (*m*/*z* 505.091; [M − H]^−^; ±2 ppm; experimental), skyrin-6-*O*-β-glucopyranoside **(9)** (*m*/*z* 699.141; [M − H]^−^; ±2 ppm; experimental), 1,2,4,5-tetrahydroxy-7-(hydroxymethyl)-9,10-anthraquinone **(10)** (*m*/*z* 301.034; [M − H]^−^; ±2 ppm; experimental), 1,2,4,5-tetrahydroxy-7-methyl-9,10-anthraquinone-2-*O*-β-glucopyranoside **(11)** (*m*/*z* 447.097; [M − H]^−^; ±2 ppm; experimental). Assigned scale bar represents 1 mm. Black insert depicts the scanned area in each case.

Dark glands of *H. annulatum* are localized on the ventral side of the leaf along with numerous leaf hairs rather than the dorsal side (Figure 5A). As anticipated, hypericin **(6)** was found to accumulate in the dark glands with higher abundances in dark glands at the leaf margins (Figure 5A). Strikingly, the distribution and localization of skyrin **(7)** were entirely dissimilar to what was observed in *H. humifusum* and *H. tetrapterum* plants. A typically scattered pattern of distribution of skyrin **(7)** was observed near or around the dark glands; however, skyrin **(7)** did not localize in the dark glands (Figure 5A). Interestingly, skyrin-6-*O*-β-glucopyranoside **(9)** accumulated in the dark glands similar to its distribution observed in *H. tetrapterum* (Figure 6; ventral leaf surface only, not dorsal). Emodin **(1)** displayed a similar pattern distribution as that of skyrin **(7)** (Figure 5A). It is well-known that in plants, secondary metabolites synthesized at a particular site are distributed across different parts of the tissues during adverse conditions such as biotic and abiotic stresses [29,30]. Hence, it might be possible that in these *Hypericum* species, skyrin **(7)** was produced in the dark glands and later translocated into the surrounding leaf tissues. Emodin anthrone **(2)** also exhibited a scattered distribution pattern around the dark glands, similar to emodin **(1)** (Figure 5A and Figure 6). Furthermore, the analogs of hypericin **(6)**, namely pseudohypericin **(4)**, protopseudohypericin **(3)**, and protohypericin **(5)**, were all found to localize in the dark glands, corroborating the previous results [7,8,24] (Figure 6).

**Figure 7:**
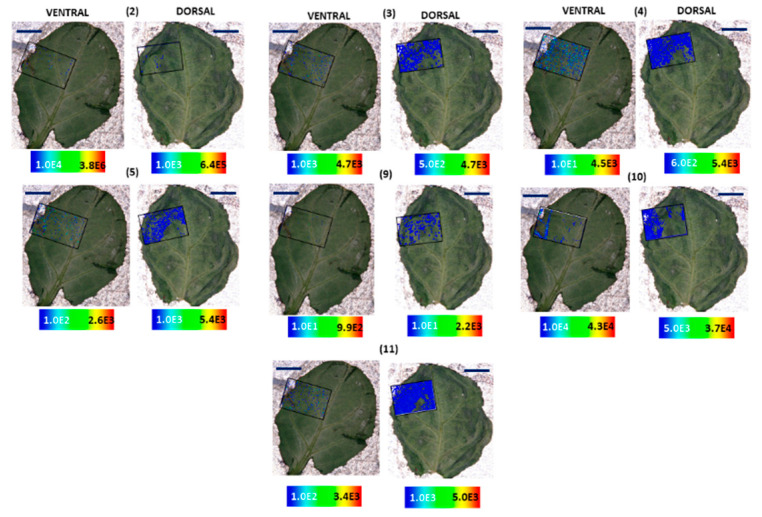
Localization and distribution of compounds in *H. bupleuroides* leaves. Emodin anthrone **(2)** (*m*/*z* 255.062; [M − H]^−^; ±2 ppm; experimental), protopseudohypericin **(3)** (*m*/*z* 521.082; [M − H]^−^; ±2 ppm; experimental), pseudohypericin **(4)** (*m*/*z* 519.066; [M − H]^−^; ±2 ppm; experimental), protohypericin **(5)** (*m*/*z* 505.091; [M − H]^−^; ±2 ppm; experimental), skyrin-6-*O*-β-glucopyranoside **(9)** (*m*/*z* 699.141; [M − H]^−^; ±2 ppm; experimental), 1,2,4,5-tetrahydroxy-7-(hydroxymethyl)-9,10-anthraquinone **(10)** (*m*/*z* 301.034; [M − H]^−^; ±2 ppm; experimental), 1,2,4,5-tetrahydroxy-7-methyl-9,10-anthraquinone-2-*O*-β-glucopyranoside **(11)** (*m*/*z* 447.097; [M − H]^−^; ±2 ppm; experimental). The assigned scale bar represents 1 mm. Black insert depicts the scanned area in each case.

Surprisingly, HPLC-HRMS analysis of *H. bupleuroides* revealed the absence of all target compounds except emodin anthrone **(2)** and skyrin **(7)**. Kucharíková et al. (2016) reported the absence of emodin **(1)**, hypericin **(6)**, and its analogs in *H. bupleuroides* [26]. Besides, our present observations were in partial agreement with our earlier results [17] in which emodin **(1)** was detected, whereas emodin anthrone **(2)** could not be detected. In our MALDI-HRMS imaging analyses, we were not able to detect intensities of compounds on the ventral side of the leaf (<LOD), except in the leaf veins where emodin **(1)** was observed (Figure 5B). Whereas, when imaging from the dorsal side, skyrin **(7),** emodin **(1)**, and hypericin **(6)** were observed in low intensities (Figure 5B and Figure 7). 

**Figure 8:**
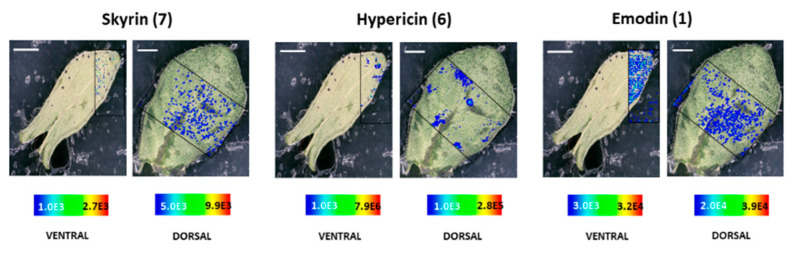
Selected ion images depicting the localization of skyrin **(7)** (*m*/*z* 537.086; [M − H]^−^; ±2 ppm; experimental), hypericin **(6)** (*m*/*z* 503.074; [M − H]^−^; ±2 ppm; experimental), and emodin **(1)** (*m*/*z* 269.045; [M − H]^−^; ±2 ppm; experimental). Occurrence and localization of skyrin **(7)**, hypericin **(6)**, and emodin **(1)** mapped from the ventral and dorsal surfaces of *H. rumeliacum* leaves. The assigned scale bar represents 1 mm. Black insert depicts the scanned area in each case.

**Figure 9:**
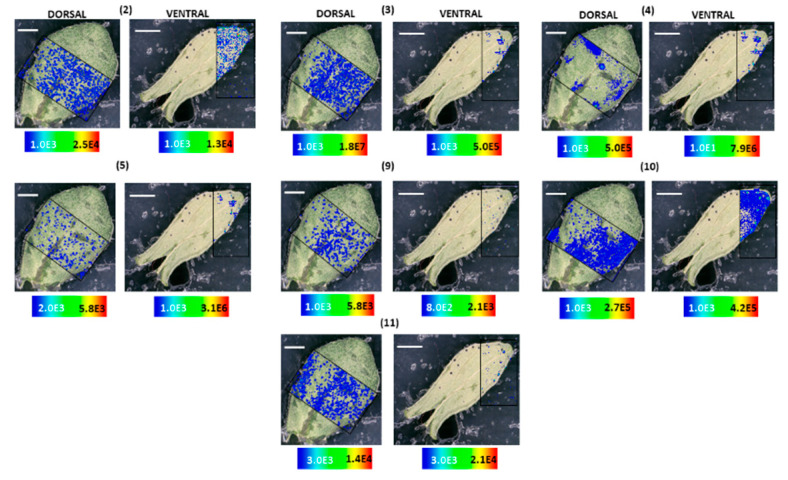
Localization and distribution of compounds in *H. rumeliacum* leaves. Emodin anthrone **(2)** (*m*/*z* 255.062; [M − H]^−^; ±2 ppm; experimental), protopseudohypericin **(3)** (*m*/*z* 521.082; [M − H]^−^; ±2 ppm; experimental), pseudohypericin **(4)** (*m*/*z* 519.066; [M − H]^−^; ±2 ppm; experimental), protohypericin **(5)** (*m*/*z* 505.091; [M − H]^−^; ±2 ppm; experimental), skyrin-6-*O*-β-glucopyranoside **(9)** (*m*/*z* 699.141; [M − H]^−^; ±2 ppm; experimental), 1,2,4,5-tetrahydroxy-7-(hydroxymethyl)-9,10-anthraquinone **(10)** (*m*/*z* 301.034; [M − H]^−^; ±2 ppm; experimental), 1,2,4,5-tetrahydroxy-7-methyl-9,10-anthraquinone-2-*O*-β-glucopyranoside **(11)** (*m*/*z* 447.097; [M − H]^−^; ±2 ppm; experimental). The assigned scale bar represents 1 mm. Black insert depicts the scanned area in each case.

The spatial distribution of compounds in *H. rumeliacum* was found to be typically corresponding to that of *H. annulatum*. Hypericin **(6)** and its analogs were found to localize in the dark glands (Figure 8 and Figure 9). Skyrin **(7)** and its precursor skyrin-6-*O*-β-glucopyranoside **(9)** were found to accumulate near the dark glands, whereas the abundance of skyrin-6-*O*-β-glucopyranoside **(9)** was high in dark glands (Figure 8 and Figure 9). Further, emodin **(1)** and emodin anthrone **(2)** were observed around the dark glands (Figure 8 and Figure 9).

## 3. Materials and Methods 

### 3.1. Plant Material and Growth Conditions

For the experiments, 5 different in vitro grown *Hypericum* species in the vegetative stage of development were used. The stock cultures of *H. humifusum* L., *H. bupleuroides* Stef., *H. annulatum* Moris L., *H. tetrapterum* Fr., and *H. rumeliacum* Boiss. were derived from seeds obtained through the *Index Seminum* exchange program and characterized by DNA barcoding [12, Bruňáková et al. unpublished]. The shoot cultures were cultivated in solid MS media (Duchefa Biochemie, Haarlem, Netherlands) containing a 4.4 g L^−1^ salt mixture according to Murashige and Skoog [31] with Gamborg’s B5 vitamins [32], 30 g L^−1^ sucrose (CentralChem, Banská Bystrica, Slovakia), 7 g L^−1^ agar (REMI M. B., Proseč nad Nisou, Czech Republic), and 2 mg L^−1^ glycine with pH adjusted to 5.65 before autoclaving. The cultures were grown at 23 ± 2 °C temperature under 16/8 h photoperiod at 90 μmol m^−2^ s^−1^ artificial irradiance. The subculture interval was 5 to 6 weeks.

### 3.2. Extraction of Metabolites From Leaves

The extraction of aboveground tissues of *Hypericum* species was performed according to our previously established procedures [7].

### 3.3. HPLC-HRMS Instrumentation and Measurement Conditions

The extracts were analyzed using an HPLC instrument (Agilent 1200 series, Santa Clara, CA, USA) coupled with LTQ Orbitrap XL mass spectrometer (Thermo Scientific, Waltham, MA, USA) with HESI (Heated electrospray interface) source. The column used was Luna C_18_ (50 × 3 mm, 3 µm particle size) from Phenomenex, USA, and oven temperature was maintained at 33 °C. Measurement parameters were according to our previously established protocol [7], slightly modified. Briefly, mobile phase was a gradient of water, 10 mmol L^−1^ ammonium acetate, and 0.1% formic acid (A) and acetonitrile and 10% methanol (B); the gradient method was as follows: 0 min, 75% A, 25% B; 0.5 min, 75% A, 25% B; 3 min, 45% A, 55% B; 5 min, 0% A, 100% B; 9 min, 0% A, 100% B; 9.1 min, 75% A, 25% B; 13 min, 75% A, 25% B; 14 min, 75% A, 25% B. The flow rate was maintained at 0.6 mL/min. The mass spectrometer was run in negative mode with a mass range of *m*/*z* 110–800 at a resolution of 60,000 at *m/z* 200. For the HRMS^n^ measurements, collision-induced dissociation was kept at 35 eV. MS^2^ measurements of skyrin **(7)** ([M − H]^−^ were executed at *m*/*z* 537.08  ±  0.5 amu with a scan range of *m*/*z* 450–600, higher-energy collisional dissociation (HCD) with 45 eV, and a resolution of 35,000 at *m*/*z* 200. The analyses were performed using Xcalibur software v. 2.2 SP1.48. (Thermo Scientific, Bremen, Germany). The detection and identification of the compounds were performed according to our previously established method [33].

### 3.4. Sample Preparation for MALDI-HRMS Imaging

Fresh leaves were harvested from healthy plants and subjected to sample preparation. In each case, the second set of 2 leaves from the shoot apex was harvested and used for analysis from both the ventral and dorsal sides. Leaves were fixed on glass slides using adhesive tapes. The samples were sprayed uniformly with matrix HCCA (alpha-cyano-4-hydroxycinnamic acid; 7 mg/mL) prepared in a 1:1 ratio of acetonitrile and distilled water with 0.1% FA. A SMALDI Prep spray device (TransMIT GmbH, Giessen, Germany) was utilized for matrix spraying. Before proceeding with MALDI-HRMS imaging, a photographic image was taken for each sample using a specialized digital microscope (VHX-5000, Keyence Deutschland GMBH, Neu-Isenburg, Germany) to evaluate the measured area and record the optical image.

### 3.5. MALDI-HRMS Imaging

MALDI-HRMS imaging experiments of the leaf samples were carried out with an atmospheric pressure scanning microprobe matrix-assisted laser desorption/ionization source (AP-SMALDI; TransMIT GmbH, Giessen, Germany) coupled with a Q-Exactive high-resolution mass spectrometer (Thermo Scientific Inc., Bremen, Germany). The parameters used were according to our previously established protocol [7], with slight modifications. A 60 Hz pulsed N_2_ laser MNL 100 series (LTB Lasertechnik GmbH, Berlin, Germany) was used for the UV beam generation at 337.1 nm. The resolution of measurement was adjusted to 10–15 μm, and measurements were made in full scan negative ion mode at *m*/*z* 100–800 mass range with an internal lock mass correction utilizing *m/z* 333.08808, corresponding to the HCCA matrix ion signal [2M−H−CO_2_]^−^. Furthermore, measurements were performed with a mass resolution of 140,000 at *m/z* 200, and the source spray voltage was set at 3000 V. For HRMS^2^ measurements in the imaging mode, the isolation width of *m*/*z* 1.5 and collision energy of 50 eV was used. HRMS^2^ measurements for the skyrin **(7)** were recorded within a mass range of *m*/*z* 500–800 in the negative-ion mode. Processing of data and mapping of mass pixels of the target compounds was done with the software package ImageQuest (v. 1.1.0; Thermo Fisher Scientific, Bremen, Germany). Ion images were generated with a bin width of ±2.0 ppm for full scans. The mass pixels are shown color-coded (Figure 2, Figure 3, Figure 4, Figure 5, Figure 6, Figure 7, Figure 8 and Figure 9), starting with blue, indicating lower intensities and red, indicating the highest intensities.

## 4. Conclusions and Outlook

Herein we report for the first time the spatial distribution of skyrin **(7)** in leaves of five *Hypericum* species using MALDI-HRMS imaging. We also mapped the localization of its precursors, namely, skyrin-6-*O*-β-glucopyranoside **(9)**, 1,2,4,5-tetrahydroxy-7-methyl-9,10-anthraquinone-2-*O*-β-glucopyranoside **(11)**, and 1,2,4,5-tetrahydroxy-7-(hydroxymethyl)-9,10-anthraquinone **(10)**. In our HPLC-HRMS and MALDI-HRMS imaging analyses, we could not detect oxyskyrin-6-*O*-β-glucopyranoside **(8)** (<LOD), probably due to the unstable behavior of the compound or that it is a reactive intermediate in the biosynthetic pathway of hypericin **(6)**. In the leaves of *H. humifusum* and *H. tetrapterum* plants, skyrin **(7)** and its precursors are localized in the dark glands along with hypericin **(6)**. In *H. annulatum*, *H. bupleuroides*, and *H. rumeliacum*, skyrin **(7)** is distributed around the dark glands similar to emodin **(1)**, whereas its precursor skyrin-6-*O*-β-glucopyranoside **(9)**, interestingly, accumulates in the dark glands. Antimicrobial activities of skyrin **(7)** further reinforce the fact that the distribution of skyrin **(7)** across tissues for combating the pathogen effects is an ecologically-primed possibility [19,20]. Besides, the present results lend a scientific handle to support further that skyrin **(7)** is an immediate precursor of hypericin **(6)** (Figure 1) due to their typical colocalization in the dark glands. Emodin **(1)** accumulation in all *Hypericum* species irrespective of hypericin **(6)** production supports the possible role of skyrin **(7)** in hypericin **(6)** biosynthesis. 

Skyrin **(7)** and its precursors are not abundantly available in plants, but these compounds are produced by different classes of endophytic filamentous fungi [34]. It could be possible that *Hypericum* plant-associated endophytes produce these metabolites in planta and contribute to hypericin production, given that endophytes are known to produce secondary metabolites found in their host plants [23,35]. Besides, native endophytes might have acquired the skyrin **(7)** producing gene machinery through horizontal gene transfer in the course of co-evolution with *Hypericum* host plants. It would be interesting to identify candidate genes responsible for converting skyrin **(7)** to hypericin **(6)**, and their biological validation would bring more light into understanding the final steps in the biosynthetic pathway of hypericin **(6)**.

## Supplementary Materials

The followings are available online. Table S1. The phytochemical composition of leaves of the five *Hypericum* species under study by HPLC-HRMS.
