# Supplementary material for: MALDI-HRMS Imaging Maps the Localization of Skyrin, the Precursor of Hypericin, and Pathway Intermediates in Leaves of Hypericum Species
Source: Molecules. 2020 Aug 31;25(17):3964. doi: 10.3390/molecules25173964 (PMC7504759; doi:10.3390/molecules25173964)
Supplement: Supplementary file 1 [file molecules-25-03964-s001.pdf]

Table S1. The phytochemical composition of leaves of the 5 *Hypericum* species by HPLC-HRMS. Selected mass ion intensities of emodin (**1**)**,** emodin anthrone **(2),** protopseudohypericin **(3),** pseudohypericin **(4),** protohypericin **(5),** hypericin **(6),** skyrin **(7),** oxyskyrin-6-*O*-β-glucopyranoside **(8)**, skyrin-6-*O*-β-glucopyranoside **(9)**, 1,2,4,5-tetrahydroxy-7-(hydroxymethyl)-9,10-anthraquinone **(10)**, and 1,2,4,5-tetrahydroxy-7-methyl-9,10-anthraquinone-2-*O*-β-glucopyranoside **(11)** are shown; n.d. = not detected or below the limit of detection (<LOD); LOQ = limit of quantitation.

**Species**	**1**	**2**	**3**	**4**	**5**	**6**	**7**	**8**	**9**	**10**	**11**
H. humifusum	9.50E+04	<LOQ	2.26E+06	n.d.	1.06E+06	7.45E+05	2.05E+05	n.d.	n.d.	2.76E+03	1.70E+05
H. bupleuroides	n.d.	1.32E+07	n.d.	n.d.	<LOQ	n.d.	1.13E+04	n.d.	n.d.	n.d.	n.d.
H. annulatum	1.94E+04	5.29E+04	2.91E+06	1.33E+06	1.08E+06	1.10E+06	1.91E+05	n.d.	n.d.	1.55E+05	4.17E+05
H. tetrapterum	2.07E+04	<LOQ	4.61E+05	1.00E+06	1.66E+05	5.42E+05	2.36E+05	n.d.	n.d.	n.d.	2.43E+05
H. rumeliacum	1.75E+05	n.d.	1.01E+06	5.76E+05	8.66E+05	3.77E+05	1.10E+05	n.d.	n.d.	1.53E+05	1.10E+05
